# Decreased P27 protein expression is correlated with the progression and poor prognosis of nasopharyngeal carcinoma

**DOI:** 10.1186/1746-1596-8-212

**Published:** 2013-12-20

**Authors:** Qingping Jiang, Huiling Yang, Chao Cheng, Hanzhen Xiong, Shaoyan Liu, Jie Long, Yajie Zhang, Weiyi Fang, Zhen Liu

**Affiliations:** 1 Department of Pathology, the Third Affiliated Hospital, Guangzhou Medical University, Guangzhou 510150, PR China; 2 Department of Pathology, Basic school, Guangzhou Medical University, Guangzhou 510000, PR China; 3 Cancer Research Institute, Southern Medical University, Guangzhou 510515, PR China; 4 School of Pharmacy, Guangdong Medical College, Dongguan 523808, PR China; 5 Pediatric Center, Southern Medical University Zhujiang Hospital, Guangzhou 51000, PR China

**Keywords:** p27, NPC, Prognosis

## Abstract

**Background:**

To determine the correlation of cyclin-dependent kinase inhibitor 1B (p27) expression with clinicopathologic features in nasopharyngeal carcinoma (NPC), including patient prognosis.

**Methods:**

Real-time PCR and immunohistochemistry were used to examine the mRNA and protein expressions of p27 in NPC and nasopharyngeal tissues. The relationship of p27 expression levels with clinical features and prognosis of NPC patients was analyzed.

**Results:**

The expression level of p27 mRNA was markedly lower in NPC tissues than that in the nasopharyngeal tissues (P = 0.0006). Specific p27 protein staining by immunohistochemistry was found in the nuclei and cytoplasm of nasopharyngeal and malignant epithelial cells but decreased expression was observed in NPC samples compared to normal epithelium samples (P = 0.002). In addition, low levels of p27 protein were inversely correlated with the status of T classification (p = 0.002) and clinical stage (p = 0.019) of NPC patients. Patients with lower p27 expression had a significantly shorter overall survival time than did patients with high p27 expression. Multivariate analysis suggested that the level of p27 expression was not an independent prognostic indicator (p = 0.682) for NPC survival.

**Conclusion:**

Low level of p27 expression is a potential unfavorable prognostic factor for patients with NPC.

**Virtual slides:**

The virtual slide (s) for this article can be found here: http://www.diagnosticpathology.diagnomx.eu/vs/1915282782109343.

## Background

Nasopharyngeal carcinoma (NPC) is a squamous cell carcinoma derived from mucosal epithelium of the nasopharynx correlated with EBV infection, environmental factors and genetic aberrance. Among them, EBV infection is the most important for NPC, when other cancers in upper digestive tract have something with to do with it [1]. The initiation and progression of NPC is a complex and consecutive process with the participation of many important genes [2-5]. In a previous study, we used cDNA microarray to detect differentially expressed genes among NPC and nasopharyngeal tissues (NP) and observed that expression of p27, a gene encoding cyclin-dependent kinase inhibitor 1B, was indicated to be significantly reduced in NPC tissues and cells, hinting its potentially suppressive role in NPC pathogenesis [6] (Additional file 1: Figure S1).

P27 is an enzyme inhibitor that in humans is encoded by the CDKN1B gene. It encodes a protein which belongs to the Cip/Kip family of cyclin dependent kinase (Cdk) inhibitor proteins. In previous investigations, down-expression of P27 had been indicated in many tumor types, including lung cancer [7], hepatocellular carcinoma [8], gastric cancer [9], colorectal cancer [10] et al., which induced the progression and poor prognosis of these tumors. Further, p27 was observed to act as a significant role in suppressing tumor pathogenesis [11,12]. Based on the above reports mentioned, p27 was taken as an important gene participating in tumor pathogenesis. The role p27 plays in NPC has also been reported in several references [13,14].p27 was found the low expression level may contribute to the aggressive behavior of NPC by immunohistochemistry.

To further clarify its role in NPC pathogenesis, the present study was to investigate the expression status of mRNA and protein in much more NPC cases and NP cases than those of the previous literatures, and then analyze the potential associations of this protein with clinicopathological features and overall survival rate in 130 cases of NPC patients.

## Materials and methods

### Cell culture

Seven (7) NPC cell lines including 5-8 F, 6-10B, CNE2, C666-1, HONE1, HNE1, and SUNE1 were maintained in RPMI 1640 supplemented with 10% newborn calf serum (NBCS) (PAA Laboratories, Inc, Austria).

### Sample collection

Sixty-one (61) primary fresh NPC samples and 20 non-cancerous fresh nasopharyngeal samples were collected at the time of diagnosis before any therapy at the People’s Hospital of Zhongshan City, China from 2012 to 2013. All fresh samples were immediately preserved in liquid nitrogen. Thirty-five (35) NP tissues and 130 undifferentiated NPC paraffin-embedded specimens with clinic and prognosis information from 2003 to 2006 were obtained from the People’s Hospital of Zhongshan City, and their age ranging from 21 to 80 years (median, 49.6 years). Brief clinical data of these NPC cases were given in Table 1. For the use of these clinical materials for research purposes, prior consent from the patients and approval from the Ethics Committees of these two hospitals were obtained. All specimens had confirmed pathological diagnosis and were staged according to the 1997 NPC staging system of the UICC.

**Table 1:** Correlation between the clinicopathologic characteristics and expression of p27 protein in NPC

**Characteristics**	**n**	**p27 (n)**		***P***
		**High expression**	**Low expression**	
Gender				
Male	93	49	44	
Female	37	13	24	0.082
Age(y)				
≥50	62	30	32	
<50	68	32	36	1.000
Smoking				
Yes	32	21	11	
No	98	41	57	0.025
Recurrence				
Yes	4	2	2	
No	126	60	66	1.000
T classification				
T_1_-T_2_	90	51	39	
T_3_-T_4_	40	11	29	0.002
N classification				
N_0_-N_1_	73	38	35	
N_2_-N_3_	57	24	33	0.291
Distant metastasis				
Yes	10	5	5	
No	129	57	63	1.000
TNM Clinical stage				
I~II	49	30	19	
III~IV	81	32	49	0.019

### Real-time PCR

Real-time PCR was used to measure the expression of p27 mRNA in fresh NPC cells, NPC tissues, and NP tissues using SYBR Premix Ex Taq (Takara, Japan) with an Mx3000P real-time PCR system (Stratagene, La Jolla, CA, USA) and the reaction was repeated three times. The sequence for sense primer was 5′- GAAGCCTGGCCTCAGAAGAC-3′, and for antisense primer was 5′- TCCAACGCTTTTAGAGGCAGAT-3′. ARF gene was used as an internal control [3].

### Immunohistochemistry

Immunohistochemistry was utilized to examine the protein expression of p27 with rabbit anti-human p27 antibody (1:100) (Santa Cruz Biotechnology, USA) according to the previous description [2]. Sections were visualized with DAB and counterstained with hematoxylin, mounted in neutral gum, and analyzed using a bright field microscope.

### Evaluation of staining

The immunohistochemically stained tissue sections of p27 in cytoplasm were reviewed and scored separately by two pathologists blinded to the clinical parameters. Expression of p27 in the nucleus and in the cytoplasm was independently evaluated and scored as previously described [12]. The sum of the cytoplasm and nuclear staining scores was used as the final staining score for p27. For statistical analysis, a final staining score of 0–6 or 7–12 was respectively considered to be low or high expression.

#### Statistical analyses

All statistical analyses were performed using SPSS 13.0 and Graphpad softwares. Data were presented as mean ± SD. Two-tailed Student’s t test was used for comparisons of 2 independent groups. The χ^2^ test was used to analyze the relationship between the levels of p27 expression and clinicopathologic characteristics. Survival curves were plotted using the Kaplan-Meier method and compared using the log-rank test. The significances of various variables in survival were analyzed using multivariate cox proportional hazards model. A P value of less than 0.05 was considered statistically significant.

## Results

### P27 mRNA is expressed at low levels in NPC tissue

**Figure 1:**
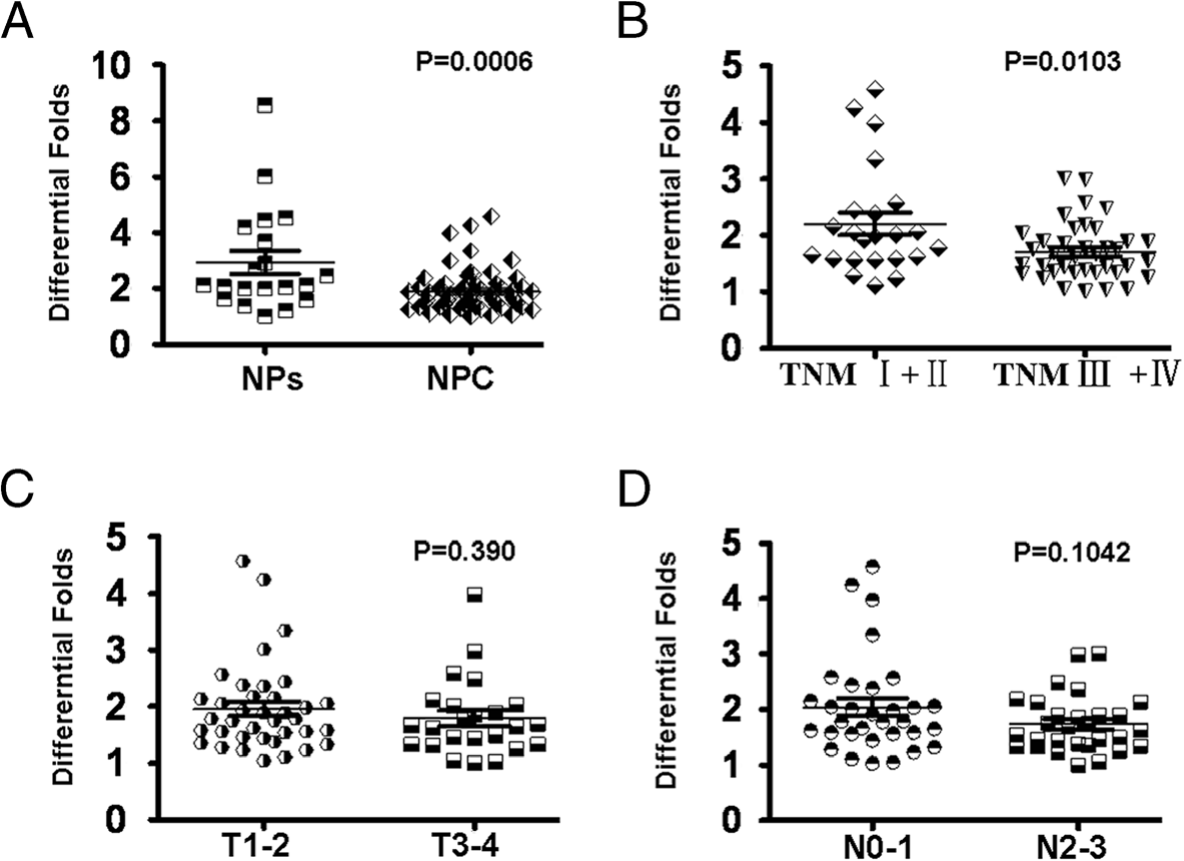
**Expression of P27 mRNA in NPC tissues and NP tissues. A**. mRNA expression of p27 was decreased in NPC tissues compared with NP tissues by real-time PCR assay. **B**. A significant reduction of p27 mRNA expression was indicated in NPC clinical stage III-IV compared with clinical stage I-II. **C**,**D**: Significantly differential mRNA expression of p27 was not observed in NPC T3-4 and N2-3 compared with T1-2 and N0-1.

In order to assess the role of p27 in NPC, we performed real-time PCR to measure the expression of p27 mRNA transcripts in 61 fresh NPC tissues and 20 fresh collected normal nasopharyngeal tissues. Compared with normal nasopharyngeal tissues, NPC cells and tissues showed reduced expression levels of p27 mRNA (p = 0.0006) (Figure 1A). Furthermore, decreased p27 mRNA expression was observed in clinical stage III-IV compared to clinical stage I-II samples (Figure 1B) (p = 0.0103). However, we did not find the significantly differential expression of p27 mRNA in T3-4 and N2-3 compared to T1-2 and N0-1 (Figure 1C, D).

### Immunohistochemical analysis of p27 protein expression in NPC and nasopharynx tissues

**Figure 2:**
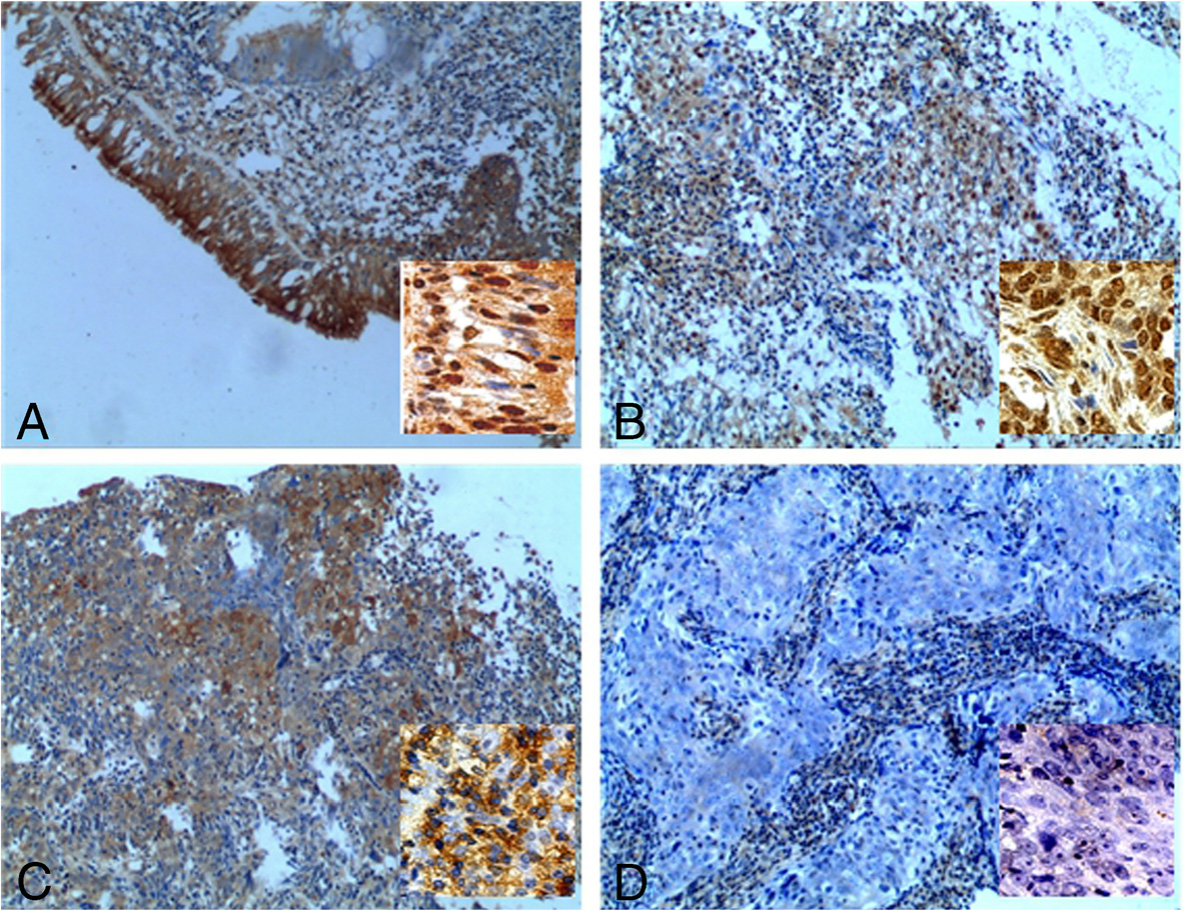
**P27 protein was expressed in the cytoplasm and nuclei of non-cancerous and malignant epithelial cells and its down-regulation was indicated in NPC tissues compared to NP samples (original magnifications are: Х 100). A** shows the strong expression of p27 in normal epithelium; and in some cases, p27 highly expresses in cytoplasm and nucleus (inset); **B** displays the high p27 expression in cytoplasm and nuclei of NPC tissues; **C** shows the positive cytoplasm p27 expression in NPC tissues; **D** indicates the negative expression of p27 in NPC tissues.(insets showing P27 expression observed under high magnification Х 400).

We measured the expression levels and subcellular localization of p27 protein in 130 archived paraffin-embedded NPC samples and 35 NP samples using immunohistochemical staining (Figure 2A-D). Specific p27 protein staining was detected in the nuclei and cytoplasm of non-cancerous and malignant epithelial cells (Figure 2A, B). Decreased expression of p27 was observed in NPC samples compared to NP tissues (P = 0.002) (Table 2).

**Table 2:** Protein expression of p27 between NPC and NP tissues

**Group**		**Protein expression(n)**		**P value**
	**Cases**	**High expression**	**Low expression**	
NPC	130	62	68	
NP tissues	35	27	8	P = 0.002

### Relationship between clinicopathological characteristics and p27 expression in NPC patients

The relationship between clinicopathological characteristics and p27 expression levels in individuals with NPC is summarized in Table 1. We did not find a significant association between p27 expression levels with patient’s age, sex, smoking, disease recurrence, N classification, and M classification in 130 NPC cases. However, we observed that the expression level of p27 was inversely correlated with tumor size (T classification) (P = 0.002) and clinical stage (I-II vs. III-IV) (P = 0.019) in NPC patients (Table 1).

### Survival analysis

**Figure 3:**
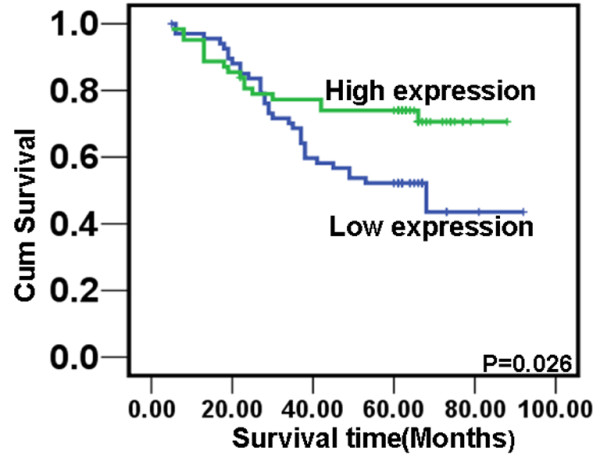
**Expression of p27 protein predicts NPC patients’ overall survival time.** Patients with high level of p27 expression had better survival than those with low level of p27 expression.

**Figure 4:**
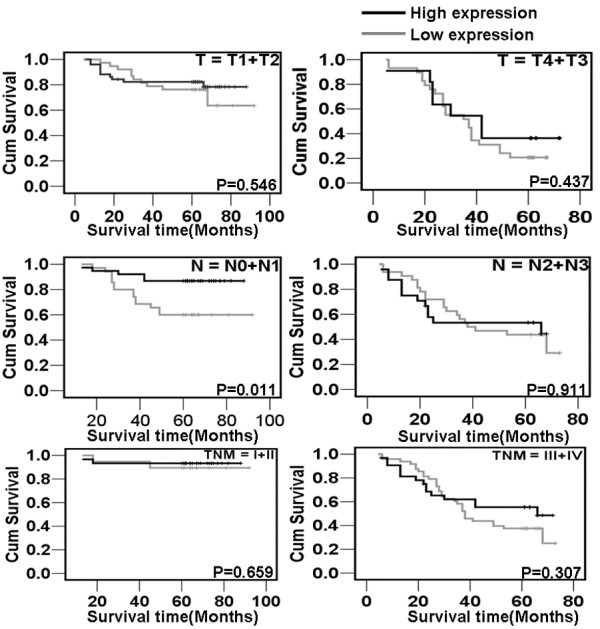
**The correlation of p27 expression with NPC patients’ survival time in strata analysis in TNM stage, T classification, N classification, and M classification.** Patients with higher expression of p27 protein indicated longer survival time only in N0-N1 classification in NPC, but not other clinical classifications.

To investigate the prognostic value of p27 expression for NPC, we assessed the association between the levels of P27 expression and patients’ survival using Kaplan–Meier analysis with the log-rank test. In 130 NPC cases with prognosis information, we observed that the level of p27 protein expression was significantly correlated with the overall survival of NPC patients (Figure 3). Patients with high levels of p27 expression had better survival than those with low levels of P27 expression (P = 0.026). In addition, we also observed that the patients with higher P27 protein expression had longer survival time N0-N1 (P = 0.011) classification (Figure 4). Univariate analyses showed that patient survival was also significantly correlated with radiotherapy, T, N, M classifications, clinical stages and the expression of p27 (P = 0.005, P<0.001, P<0.001, P<0.001, P<0.001, and P = 0.032 respectively). To determine whether p27 is an independent prognostic factor for NPC, we performed multivariate analysis of P27 protein expression levels adjusted for age, gender, smoking status, T classification, N classification, M classification, and clinical stages of NPC patients. These results showed that the level of P27 expression was not an independent prognostic factor for NPC (P = 0.682) (Table 3).

**Table 3:** Summary of Cox regression analysis of overall survival duration of P27 nuclear and cytoplasm expression

**Parameter**	**Univariate analysis**			**Multivariate analysis**		
	***P***	**HR**	**95% CI**	***P***	**HR**	**95% CI**
Gender						
Male vs. female	0.485	0.488	0.665-2.361			
Age						
≥50 vs. <50 years	0.787	0.073	0.620-1.881			
Family tumor history						
Yes vs. No	0.349	0.877	0.000-28.401			
Smoking						
Yes vs. No	0.387	0.748	0.368-1.474			
Chemotherapy						
Yes vs. No	0.700	1.153	0.560-2.372			
Radiotherapy						
Yes vs. No	0.005	0.369	0.184-0.742	0.944	1.032	0.431-2.469
T classification						
T_1_-T_2_ vs. T_3_-T_4_	0.000	13.289	1.884-8.223	0.014	6.032	1.225-6.067
N classification						
N_0_-N1 vs. N_2--_N_3_	0.000	2.950	1.662.-5.237	0.240	1.379	0.720-3.700
M classification						
M_0_ vs. M_1_	0.000	35.042	5.031-24.906	0.001	10.438	1.803-11.112
TNM Clinical stage						
I-II vs. III-IV	0.000	19.386	3.592-27.943	0.051	3.795	0.992-14.238
P27 expression						
High expression vs. low expression	0.030	4.724	0.290-0.938	0.682	0.168	0.471-1.637

## Discussion

NPC is a unique malignant head/neck cancer with remarkably distinctive ethnic and geographic distribution among the world.Though there are some other neoplasms such as lymphoma, carcinoma of salivary gland and rare melanotic disease [15], NPC is the most common in nasopharyngeal region. It is a disease with disorder of cell cycle and uncontrolled growth due to abnormal expression of cell cycle factors [4,6,16]. P27, a key cell cycle protein kinase inhibitor, formed a complex with CCND1 and CDK4 that prevents CDK4 from adding phosphate residues to its principal substrate, the retinoblastoma (pRb) protein and blocks the cell-cycle G1/S transition. Furthermore, p27 is able to bind other Cdk proteins when it connected to cyclin subunits such as Cyclin A/Cdk2 and Cyclin E/Cdk2 and suppress the transition of cell cycle. Decreased expression of p27 has been indicated in many tumors, including lung cancer [17], bladder cancer [18], melanoma [19], and ovarian cancer [20], prostate cancer [21], which was associated with the unfavorable clinical parameters and poor outcomes of these tumors. However, the role of p27 is still unclear in NPC.

In a previous investigation, we found the significantly attenuated levels of p27 mRNA in NPC tissues and cells compared to NP tissues [6] by gene microarray analysis. In this study, we validated the significantly differential expression of mRNA in NPC tissues and NPC cells compared to NP tissues by real-time PCR. Our reports were similar with the investigation of p27 mRNA expression in other tumors [17-19]. Furthermore, we also found that reduced mRNA p27 expression was shown in clinical stage of III-IV compared to clinical stage of I-II. These results preliminarily suggested p27 as a potentially suppressive gene involved in the pathogenesis of NPC.

In subsequent exploration, we observed that p27 protein was a co-expression factor of cytoplasm and nucleus in NPC tissues and nearly a half of NPC cases showed nuclear expression of p27. In line with the mRNA expression examination, p27 protein expression was significantly down-regulated in NPC tissues compared to NP tissues. This result further provided evidence to support p27 as a candidate suppressor in NPC. Further, we analyzed the correlation of p27 protein expression with clinical features of NPC patients. We found that although p27 protein expression was not associated with patient’s age, sex, smoking, N classification (lymph node metastasis) or M classification (distant metastasis), it was positively correlated with T classification (tumor size) and clinical stage. This result was similar to our report of p27mRNA expression of in NPC. Furthermore, we presented the evidence that protein expression of p27 protein in NPC was inversely correlated with patient’s overall survival time. Patients with high protein expression of p27 had an overall longer survival time. Our results here suggest that p27 protein expression is taken as a tumor suppressor inhibiting the pathogenesis of NPC.

Furthermore, survival prognosis was assessed by stratification analysis against different T classification, N classification, M classification, and clinical stage. We observed that p27 protein expression was only positively associated with the survival time for NPC patients in N0-1 classification. Patients with high p27 expression had better prognosis than those of negative nuclear expression. These results hinted that p27 expression is a good biomarker for evaluating the prognosis of NPC patients with N0-1 classification.

Finally, we evaluate the possibility of p27 protein expression as an independent prognostic factor. According to univariate analysis, patient’s overall survival is inversely proportional to T/N/M classification, clinical stage, but positively correlated with p27 protein expression and radiotherapy. Multivariate analyses showed that nuclear expression of p27 protein was not an independent predictor of prognosis for NPC patients regardless of its patients’ disease status. This result was not consistent with the reports from other tumors including breast cancer [22] and glioma [23] etc.

In summary, our results provide evidence that expression of p27 may be involved in the clinical progression and poor prognosis of NPC patients. However, it could not serve as a potential independent prognostic factor for NPC patients.

## Conclusion

In summary, our study demonstrated that the expression level of p27 was significantly reduced in NPC and correlated with the malignant status and poor prognosis for NPC patients. Due to the limited sample size of patients in our investigation, further studies are needed to verify these findings and establish the role of p27 as a reliable clinical predictor for the outcome of NPC patients.

## Abbreviations

P27: Cyclin-dependent kinase inhibitor 1B; NPC: Nasopharyngeal carcinoma; PCR: Polymerase chain reaction.

## Competing interests

The authors declare that they have no competing interests.

## Authors’ contributions

QP J designed the whole experiment. HL Y and C C made contributions to acquisition of clinical data. HZ X collected biopsy tissues and archive blocks. SY L carried out immunhistochemical staining and LJ Real-time PCR. YJ Z and WY F drafted the manuscript. ZL revised manuscript critically for important intellectual content and had given final approval of the version to be published. All authors read and approved the final manuscript.

## Supplementary Material

Additional file 1: Figure S1 Reduced expression of p27 in NPC tissues and cells compared to NP tissues by microarray analysis. Click here for file
