# Supplementary figures and images for: Decreased P27 protein expression is correlated with the progression and poor prognosis of nasopharyngeal carcinoma
Source: Diagn Pathol. 2013 Dec 20;8:212. doi: 10.1186/1746-1596-8-212 (PMC3878167; doi:10.1186/1746-1596-8-212)

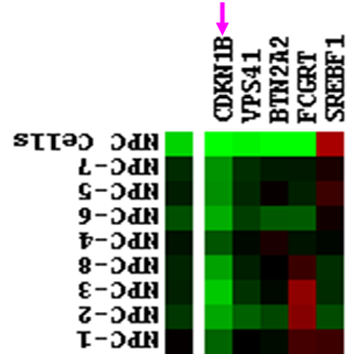

Supplement: Additional file 1: Figure S1 — Reduced expression of p27 in NPC tissues and cells compared to NP tissues by microarray analysis. [file 1746-1596-8-212-S1.tiff]
